# Nephrotic Range Proteinuria in Preeclampsia Associated With Adverse Pregnancy Outcomes in Comparison to Non-nephrotic Range Proteinuria: A Prospective Cohort Study

**DOI:** 10.7759/cureus.90825

**Published:** 2025-08-23

**Authors:** Shrayasi Ghosh, Parvathi Tejanaik, Haritha Sagili, Zachariah Bobby

**Affiliations:** 1 Obstetrics and Gynaecology, All India Institute of Medical Sciences, New Delhi, IND; 2 Obstetrics and Gynaecology, Jawaharlal Institute of Postgraduate Medical Education and Research, Puducherry, IND; 3 Biochemistry, Jawaharlal Institute of Postgraduate Medical Education and Research, Puducherry, IND

**Keywords:** eclampsia, maternal outcome, nephrotic range proteinuria, perinatal outcome, pre-eclampsia

## Abstract

Aims and objectives: To compare pregnancy outcomes among pre-eclamptic women with nephrotic and non-nephrotic range proteinuria.

Methods: This prospective cohort study was conducted from May 2021 to December 2022 at a tertiary care university hospital in South India. We included a total of 278 women diagnosed with preeclampsia, 56 with and 222 without nephrotic range proteinuria in a 1:4 ratio, respectively. The relative risks (RR) of core maternal and perinatal adverse outcomes for preeclampsia among the two groups were compared. Using logistic regression analysis, we calculated the odds ratio adjusted for maternal characteristics (aOR).

Results: The women with nephrotic range proteinuria had a higher risk of eclampsia (RR 4.29, 95% CI 2.07-8.89, p<0.01), hemolysis, elevated liver enzymes, low platelet (HELLP) syndrome (RR 6.79, 95% CI 2.8-16.46, p<0.01), abruptio placentae (RR 4.95, 95% CI 2.05-11.97, p<0.01), pulmonary oedema (RR 5.94 95% CI 2.55-13.84, p<0.01), acute kidney injury (RR 4.75 95% CI 1.5-15.02, p 0.003), retinal detachment (RR 23.78 95% CI 2.92-193.5, p <0.01), stillbirth (RR 7.48 95% CI 3.52-15.89, p<0.001), small for gestational age (RR 1.45 95% CI 1.27-1.65, p <0.001), respiratory support (RR 2.06 95% CI 1.12-3.76, p<0.02) and neonatal mortality (RR 3.3 95% CI 1.04-10.43, p 0.04) compared to non-nephrotic range proteinuria. Most of the women with nephrotic proteinuria had early-onset preeclampsia (64.28% versus 36.03%, p<0.01), increasing the risk of prematurity and neonatal morbidity and mortality.

Conclusion: In women diagnosed with preeclampsia, the nephrotic range proteinuria was associated with poor maternal and perinatal outcomes and should be considered as a predictor of adverse pregnancy outcomes.

## Introduction

Preeclampsia (PE) is a pregnancy-specific, multisystemic progressive syndrome that affects 2-8% of pregnancies worldwide [1]. It is one of the major causes of maternal and perinatal mortality and morbidity worldwide. It is characterised by hypertension with proteinuria, or hypertension with significant end-organ dysfunction with or without proteinuria after 20 weeks of gestation [2]. Impaired vascular remodelling of the placental vasculature causes underperfusion, leading to global endothelial dysfunction that affects hematologic, cardiovascular, respiratory, neural, renal, and hepatobiliary systems, resulting in multiple end-organ dysfunctions. 

Preeclampsia is the most common cause of nephrotic-range proteinuria [3]. Previously, proteinuria was quantified to assess the severity of organ dysfunction in PE. In 2013, the American College of Obstetricians and Gynaecologists (ACOG) and the International Society for Study of Hypertension in Pregnancy (ISSHP) removed proteinuria as a mandatory criterion for diagnosing PE. In the absence of proteinuria, PE can be diagnosed if features of end-organ damage are present, such as acute kidney injury, hepatic dysfunction, thrombocytopenia/haemolysis, and neurological features [4,5]. Massive proteinuria (>5g/24 hours) was also removed as a possible feature of PE with severe features [4].

The reference standard method for quantifying proteinuria is the 24-hour urine protein collection. The classic cut-off to define proteinuria during pregnancy is >300mg/24 hours. According to this definition, isolated proteinuria in pregnancy is about 8%. This value regarding adverse pregnancy outcomes has not been evidence-based [6]. Nephrotic-range proteinuria is defined as proteinuria >3.5g per 24 hours/spot urine protein-creatinine ratio (uPCR) >3.5, without clinically overt nephrotic syndrome [7]. However, in clinical practice, we have observed that the severity of proteinuria has an impact on the adverse pregnancy outcomes in women with PE.

Several studies, mostly retrospective record reviews, have concluded that the severity of proteinuria negatively impacts the composite obstetric outcome [2,8-12]. There is insufficient prospective data to answer this decade-long debate concerning proteinuria in PE and adverse pregnancy outcomes with nephrotic and non-nephrotic range proteinuria. This prospective study aimed to compare the adverse pregnancy outcomes among women with preeclampsia with nephrotic and non-nephrotic range proteinuria.

This article was previously presented as an e-poster at the RCOG World Congress 2023 on June 12-14, 2023.

## Materials and methods

Study design and setting

This prospective cohort study was conducted at the Women and Children’s Hospital of Jawaharlal Institute of Postgraduate Medical Education and Research, Puducherry, India, from May 2021 to December 2022. The Institute Ethics Committee for Observational Studies approved the study protocol (JIP/IEC/2020/290). Annually, approximately 15000 deliveries (vaginal and caesarean) are conducted in our centre. Women diagnosed with pre-eclampsia, as per ACOG 2020 guidelines [1], were included in the study after obtaining informed written consent. We excluded women with chronic hypertension, pre-existing renal disease, SLE with lupus nephritis, urinary tract infection, and pre-existing proteinuria.

A total of 278 women diagnosed with PE were prospectively recruited in a 1:4 ratio, 56 with nephrotic range proteinuria (≥3.5 grams of protein in 24-hour urine or spot uPCR ≥3.5) and 222 with non-nephrotic range proteinuria (300mg-<3.5grams of protein in 24-hour urine or spot uPCR 0.3-<3.5) [13]. Maternal data on sociodemographic, clinical and biochemical characteristics, management approaches, and maternal and perinatal outcomes were collected and compared. All hospitalised women were monitored during pregnancy and postpartum until discharge.

Maternal and perinatal outcomes studied

The core set of pregnancy outcomes for PE were compared between the groups. Maternal outcomes of interest were the proportion of eclampsia, raised liver enzymes (twice the normal concentration), thrombocytopenia (<1.5 lac platelets/µl), magnesium sulphate use, abruptio placentae, pulmonary oedema, acute kidney injury (serum creatinine>1.1 mg/dl), admission to the intensive care unit, intubation and mechanical ventilation, liver capsule hematoma or rupture, stroke, cortical blindness, retinal detachment, and maternal mortality. Perinatal outcomes like stillbirth, small for gestational age (SGA), neonatal seizures, admission to the neonatal unit, respiratory support, APGAR score <7 at five minutes and neonatal mortality were compared between groups [14].

Sample size

The sample size was calculated using OpenEpi version 3.01 (release 2013; CDC, Atlanta, GA, USA). Based on the previous study by Ozkara et al. [13], we considered eclampsia as an adverse maternal outcome in preeclampsia with nephrotic range proteinuria to be 11% compared to those with non-nephrotic range group to be 1%; the estimated sample size was 278 (56 in nephrotic and 222 in non-nephrotic group) at 95% CI, 80% power and with expected attrition of 10%, and the ratio between the two groups as 1:4.

Statistical analyses

Data was analysed using SPSS Statistics version 19.0 (IBM Corp., Armonk, NY, USA). Continuous variables are expressed as mean with standard deviation (SD) or median with range (IQR), depending on the normality of the data distribution and analysed using an independent Student's t-test or Mann-Whitney U test as appropriate. Categorical variables are expressed in proportion with a 95% confidence interval and compared using the chi-square test. Relative risk with a 95% confidence interval was calculated to compare adverse pregnancy outcomes between the two groups. Multivariate logistic regression analysis was performed to study the association of various risk factors with adverse maternal and perinatal outcomes. A p-value < 0.05 was considered significant.

## Results

The baseline maternal clinical, demographic and biochemical characteristics are presented in Table 1.

**Table 1:** Clinical demographics and biochemical characteristics of study participants Abbreviations: BMI: Body Mass Index; PE: Preeclampsia; AST: Aspartate Transaminase; ALT: Alanine Transaminase; PCR: Protein-Creatinine Ratio Values expressed as mean±SD or n (%). †Independent t-test was used. §Chi-square (χ2) test was used. *A p-value < 0.05 was considered significant.

Variables	Nephrotic range (n=56) (%)	Non-nephrotic range (n=222) (%)	Test Statistics	P Value
Age (years)	26.17± 4.66	26.96 ±5.38	0.32^†^	0.31
Parity			0.319^§^	0.58
Nulliparous	37 (66)	138 (62.16)		
Multiparous	19 (33.92)	84 (37.83)		
BMI (kg/m^2^)	31.65 ±5.48	28.74 ±4.56	11.23^†^	<0.01^*^
Gestational weight gain (kg)	8.01 ±4.69	7.13 ±3.38	0.24^†^	0.11
Onset of PE (weeks)			11.46^§^	<0.01^*^
Early onset (<34)	36 (64.28)	80 (36.03)		
Late-onset (≥34)	20 (35.71)	142 (63.96)		
Severity of PE at admission			16.51^§^	<0.01^*^
Non-severe PE	27 (48.21)	173 (77.92)		
Severe PE	29 (51.78)	49 (22.07)		
Gestational age at birth (weeks)	32 ± 3	35 ± 2	15.22^†^	<0.01^*^
Spot Urine PCR	8.22 ± 6.14	1.78 ± 2.01	16.79^†^	<0.01^*^
24-hour urine protein (mg/24h)	8014.5 ± 5656.62	792.6 ± 732.3	16.66^†^	<0.01^*^
Thrombocytopenia (<1.5lac platelets/µL)	17 (30.5)	22 (10)	15.79^§^	<0.01^*^
Altered liver enzymes (AST & ALT ≥70 IU/L)	5 (8.92)	4 (1.8)	6.55^§^	0.007^*^
Serum Creatinine (≥1.1 mg/dl)	6 (10.71)	5 (2.25)	7.60^§^	0.004^*^
Serum total protein (g/dl)	5.5± 0.72	6.00 ±0.70	-3.93^†^	0.0001^*^
Serum albumin (g/dl)	2.75± 0.40	3.01± 0.45	-3.86^†^	0.0001^*^

The mean age, parity and gestational weight gain were comparable between the two groups. Prepregnancy body mass index (BMI) was significantly higher, with a mean of 31.65 ± 5.48 kg/m2 in the nephrotic group and 28.74 ± 4.56 kg/m2 in the non-nephrotic group (p < 0.01). There was a significantly increased proportion of early-onset pre-eclampsia in the nephrotic range group (64.28%) compared to the non-nephrotic group (36.03%), p-value <0.01. More than half of the participants in the nephrotic group presented with severe PE (Blood Pressure ≥ 160/110 mm Hg) at the time of admission, whereas less than one-fourth had severe PE in the non-nephrotic group. The mean 24-hour urine protein excretion in the nephrotic range group was 8014.5 ± 5656.62 mg/24 hour (urine protein-creatinine ratio (uPCR) 8.22 ± 6.14), and in the non-nephrotic group was 792.6 ± 732.3 mg/24 hour (1.78 ± 2.01). The proportion of women with thrombocytopenia (30% vs 10%, p <0.01), altered liver enzymes (8.9% vs 1.8%, p 0.007), and elevated serum creatinine levels (10.71% vs 2.25%, p 0.004) were significantly higher in the nephrotic range group when compared to the non-nephrotic group.

The comparison of the core adverse maternal outcomes in both groups is shown in Table 2.

**Table 2:** Comparison of adverse maternal outcomes Abbreviations: HELLP: Hemolysis, Elevated Liver enzymes, and Low Platelet count; ICU: Intensive Care Unit *A p-value < 0.05 was considered significant.

Variables	Nephrotic range (n=56) (%)	Non-nephrotic range (n=222) (%)	Relative risk (95% CI)	P value
Eclampsia	13 (23.21)	12 (5.4)	4.29 (2.07-8.89)	<0.01^*^
HELLP syndrome	12 (21.42)	7 (3.15)	6.79 (2.8-16.46)	<0.01*
Abruptio Placentae	10 (17.85)	8 (3.6)	4.95 (2.05-11.97)	<0.01*
Magnesium Sulphate use	53 (94.64)	146 (65.76)	1.43 (1.28-1.61)	<0.01*
Pulmonary edema	12 (21.42)	8 (3.6)	5.94 (2.55-13.84)	<0.01*
Acute Kidney Injury	6 (10.71)	5 (2.25)	4.75 (1.5-15.02)	<0.01*
ICU admission & mechanical ventilation	3 (5.3)	4 (1.8)	2.97 (0.68-12.9)	0.12
Retinal detachment	6 (10.71)	1 (0.45)	23.78 (2.92-193.5)	<0.01*

There was higher proportion of eclampsia (RR 4.29, 95% CI 2.07-8.89, p <0.01), hemolysis, elevated liver enzymes, low platelet (HELLP) syndrome (RR 6.79, 95% CI 2.8-16.46, p <0.01), magnesium sulphate administration (RR 1.43, 95% CI 1.28-1.61, p <0.01), abruptio placentae (RR 4.95, 95% CI 2.05-11.97, p <0.01), pulmonary oedema (RR 5.94, 95% CI 2.55-13.84, p<0.01), acute kidney injury (RR 4.75, 95% CI 1.5-15.02, p <0.01) and retinal detachment (RR 23.78, 95% CI 2.92-193.5, p <0.01) compared to non-nephrotic group. The need for ICU care and mechanical ventilation was comparable in both groups. There were no cases of stroke, cortical blindness, liver capsule hematoma or rupture among either of the groups. During the study period, one maternal death occurred in a woman with nephrotic range proteinuria due to a massive intracranial haemorrhage. She was referred to our emergency services with a history of eclampsia, a Glasgow Coma Scale (GCS) of 3 on mechanical ventilation with ionotropic support. Before transferring to our centre, she was hospitalised in the nearby private medical college and was evaluated for severe preeclampsia with nephrotic range proteinuria. She had a spot uPCR of 33.5 with a 24-hour urine protein of 17120 mg. All other causes for proteinuria were evaluated. Her duration of hospital stay was less than an hour. The majority of the women in the nephrotic group underwent preterm induction of labour for either maternal or fetal indication, which was found to be statistically significant (RR 3.21, 95% CI 1.65-6.26, p <0.01). The most common indication for induction of labour in both groups was severe preeclampsia (26.5%), followed by fetal growth restriction (FGR) with absent end diastolic flow (AEDF) on umbilical artery doppler studies (4.68%). 

The core adverse perinatal outcomes in both groups are presented in Table 3.

**Table 3:** Comparison of adverse perinatal outcomes Abbreviations: SGA: Small for Gestational Age; NICU: Neonatal Intensive Care Unit *A p-value < 0.05 was considered significant.

Variables	Nephrotic Range (n=56) (%)	Non-nephrotic Range (n=222)(%)	Relative Risk (95% CI)	P value
Stillbirth	17 (30.35)	9 (4.05)	7.48 (3.52-15.89)	<0.001^*^
SGA	30 (53.57)	139 (62.61)	1.45 (1.27-1.65)	<0.001*
APGAR <7 at 5 minutes	10 (17.85)	30 (13.51)	1.82 (0.97-3.43)	0.06
NICU admission	29 (51.78)	95 (42.79)	1.6 (1.25-2.04)	0.001*
Respiratory support	13 (23.21)	25 (11.26)	2.06 (1.12-3.76)	0.02*
Neonatal death	5 (8.92)	6 (2.7)	3.3 (1.04-10.43)	0.04*

The perinatal complications like stillbirth (RR 7.48, 95% CI 3.52-15.89, p <0.001), SGA (RR 1.45, 95% CI 1.27-1.65, p<0.001), NICU admission (RR 1.6, 95% CI 1.25-2.04, p 0.001), requirement of respiratory support (RR 2.06, 95% CI 1.12-3.76, p 0.02), and neonatal mortality (RR 3.3, 95% CI 1.04-10.43, p 0.04) were significantly higher in the nephrotic group than in non-nephrotic group. Overall, the neonatal mortality in both groups was higher in women with early-onset preeclampsia, secondary to prematurity with severe FGR. The APGAR score < 7 at five minutes and neonatal seizures were comparable between the groups.

On multivariate logistic regression, the mean age, uPCR, mean platelet count and mean creatinine were independent predictors of adverse maternal outcomes like eclampsia, HELLP syndrome, abruptio placentae, pulmonary edema, and retinal detachment as presented in Table 4.

**Table 4:** Multivariate logistic regression of variables predicting adverse maternal outcomes Abbreviations: BMI: Body Mass Index; POG: Period of Gestation; BP: Blood Pressure; PCR: Protein-Creatinine Ratio; AST: Aspartate Transaminase; HELLP Syndrome: Hemolysis, Elevated Liver enzymes, Low Platelet

SL No	Variable	Eclampsia		HELLP Syndrome		Abruptio placentae		Pulmonary edema		Retinal detachment	
		Adjusted OR (95% CI)	p value	Adjusted OR (95% CI)	p value	Adjusted OR (95% CI)	p value	Adjusted OR (95% CI)	p value	Adjusted OR (95% CI)	p value
1	Mean age	0.058 (0.01-0.27)	<0.01	1.95 (0.32-11.6)	0.4	1.25 (0.29-5.33)	0.75	0.23 (0.05-0.97)	0.04	0.55 (0.01-19.6)	0.9
2	BMI	1.89 (0.91-3.90)	0.083	1.1 (0.35-3.4)	0.86	0.85 (0.39-1.81)	0.67	0.99 (0.49-1.98)	0.98	0.58 (0.15-2.18)	0.42
3	Nulliparity	0.81 (0.26-2.4)	0.71	0.80 (0.12-5.35)	0.82	0.66 (0.18-2.36)	0.52	1.55 (0.50-4.77)	0.43	1.1 (0.12-10.6)	0.90
4	POG at diagnosis	1.19 (0.45-3.16)	0.71	0.49 (0.10-2.4)	0.38	1.6 (0.55-4.66)	0.38	1.01 (0.38-2.7)	0.97	0.46 (0.06-3.31)	0.44
5	BP at admission	1.61 (0.58-4.42)	0.35	0.55 (0.08-3.74)	0.54	0.69 (0.19-2.5)	0.57	2.23 (0.74-6.72)	0.15	2.1 (0.23-19.7)	0.49
6	Urine PCR	1.06 (0.94-1.1)	0.28	1.12 (1.01-1.12)	0.02	1.14 (1.02-1.27)	0.04	1.04 (0.95-1.15)	0.33	1.37 (1.09-1.73)	0.006
7	24-hour Urine Protein	1.00 (0.99-1.00)	0.99	1.00 (0.99-1.00)	0.22	1.00 (0.99-1.00)	0.86	1.00 (0.99-1.00)	0.37	0.99 (0.99-1.00)	0.30
8	Serum total Protein	1.4 (0.68-2.9)	0.35	1.36 (0.47-3.93)	0.56	0.79 (0.31-1.99)	0.63	0.80 (0.38-2.7)	0.56	0.58 (0.11-2.96)	0.51
9	Serum albumin	0.67 (0.21-2.09)	0.49	0.63 (0.12-3.35)	0.59	0.80 (0.18-3.59)	0.78	0.55 (0.18-1.63)	0.28	0.77 (0.04-13.26)	0.85
10	Mean platelet	0.59 (0.32-1.1)	0.10	0.04 (0.01-0.20)	<0.01	1.24 (0.65-2.38)	0.50	0.45 (0.22-0.95)	0.03	0.97 (0.21-4.33)	0.97
11	Mean creatinine	5.67 (0.91-35)	0.062	34.8 (2.55-477)	<0.01	0.65 (0.04-9.62)	0.75	1.08 (0.05-20.67)	0.95	0.24 (0.002-25.4)	0.55
11	Mean AST	0.99 (0.99-1.0)	0.88	0.98 (0.96-1.00)	0.18	1.01 (0.99-1.02)	0.21	1.01 (0.99-1.03)	0.13	0.98 (0.97-1.0)	0.06

For predicting adverse perinatal outcomes, such as stillbirth, SGA, NICU admission, and neonatal mortality, independent risk factors were identified in a multivariate logistic regression model, including gestational age at diagnosis, Blood Pressure at admission, mean aspartate transaminase (AST) levels, and mean birth weight, as shown in Table 5.

**Table 5:** Multivariate logistic regression of variables predicting adverse perinatal outcomes Abbreviations: BMI: Body Mass Index; POG: Period of Gestation; BP: Blood Pressure; PCR: Protein-Creatinine Ratio; SGA: Small for Gestational Age; NICU: Neonatal Intensive Care Unit; AST: Aspartate Transaminase

Sl. No.	Variable	Stillbirth		SGA		NICU admission		Neonatal mortality	
		Adjusted OR (95% CI)	p value	Adjusted OR (95% CI)	p value	Adjusted OR (95% CI)	p value	Adjusted OR (95% CI)	p value
1	Age	0.52(0.11-2.39)	0.4	0.49(0.22-1.06)	0.07	1.62(0.66-3.97)	0.28	0.52(0.06-4.01)	0.53
2	BMI	0.87(0.36-2.09)	0.76	0.70(0.46-1.07)	0.1	1.31(0.78-2.2)	0.29	0.51(0.17-1.49)	0.22
3	Nulliparity	2.39(0.72-7.91)	0.15	1.3(0.63-2.65)	0.46	1.37(0.60-3.12)	0.45	1.48(0.33-6.5)	0.59
4	POG at diagnosis	8.2(.05-1162.1)	0.05	18.07(7.09-46.04)	<0.01	3.01(1.26-7.18)	0.01	4.24(0.31-56.3)	0.27
5	BP at admission	0.88(0.27-2.85)	0.83	2.57(1.12-5.88)	0.02	1.42(0.57-3.50)	0.44	1.12(0.23-5.38)	0.88
6	Urine PCR	1.17(0.98-1.41)	0.07	0.98(0.85-1.12)	0.79	0.98(0.83-1.17)	0.89	1.13(0.87-1.46)	0.35
7	24-hour urine protein	1.0(0.99-1.0)	0.44	1.0(0.99-1.00)	0.08	1.00(0.99-1.00)	0.53	0.99(0.99-1.00)	0.37
8	Serum total Protein	0.93(0.26-3.27)	0.92	0.80(0.47-1.38)	0.43	0.89(0.48-1.64)	0.71	1.39(0.45-4.22)	0.55
9	Serum albumin	1.43(0.16-12.7)	0.74	0.88(0.40-1.92)	0.75	0.95(0.37-2.39)	0.91	0.40(0.07-2.31)	0.31
10	Mean platelet	0.86(0.45-1.61)	0.64	0.91(0.61-1.35)	0.65	1.10(0.71-1.71)	0.66	1.06(0.47-2.36)	0.87
11	Mean AST	1.05(1.01-1.10)	0.01	0.99(0.99-1.00)	0.43	1.00(0.96-1.04)	0.74	0.94(0.85-1.04)	0.29
12	Mean Birth Weight	0.007(0.004-0.117)	<0.01	-	-	0.06(0.02-0.15)	<0.01	0.195(0.03-1.00)	0.05

In the subgroup analysis among women with early-onset pre-eclampsia (onset of pre-eclampsia before 34 weeks of gestation), we compared the core adverse pregnancy outcomes. At the time of admission, there were 36 (64.28%) participants in the nephrotic range group and 80 (36.03%) in the non-nephrotic range group with early-onset pre-eclampsia. The maternal outcomes, like eclampsia, HELLP syndrome, abruptio placentae, magnesium sulphate use, and pulmonary edema, and perinatal outcomes like stillbirth and neonatal mortality, were significantly higher in the nephrotic range group compared to the non-nephrotic range proteinuria group. Acute kidney injury, ICU admission, APGAR score, NICU admission and neonatal seizures were comparable between the groups.

## Discussion

The ACOG recommendation for diagnosing PE no longer considers proteinuria an essential criterion, and 24-hour proteinuria ≥ 5g was removed as a diagnostic criterion for severe PE [1,4]. End-organ damage was included as one of the diagnostic criteria. Many obstetricians and researchers worldwide still consider that the severity of proteinuria is associated with adverse pregnancy outcomes.

Our study results showed an increased proportion of the core adverse maternal and perinatal outcomes in women with nephrotic range proteinuria than in non-nephrotic range proteinuria. The new onset of nephrotic range proteinuria with hypertension could be a marker for the early onset of disease and the need for early termination of pregnancy. In our results, we found that more than half of the women with nephrotic range proteinuria had early onset PE, with severe PE requiring premature termination of pregnancy.

Most of the individual maternal outcomes, like eclampsia, HELLP syndrome, abruptio placentae, retinal detachment, pulmonary edema, and magnesium sulphate requirement, were significantly higher in the nephrotic range proteinuria group than in the non-nephrotic range proteinuria group in our study. Other similar studies showed statistically significant composite maternal adverse outcomes, but individual adverse outcomes were not statistically significant [3,6,7,9,10,15-17]. This can likely be explained by the greater number of primigravida, higher mean BMI, and early onset preeclampsia in our study compared to other studies. Eclampsia was the most common complication encountered among our women with nephrotic-range proteinuria. A similar observation was seen by Li et al. in women with ≥5g/24 h urine protein and ≥3001 mg/24-hour proteinuria group in the study by Ozkara et al. [8,14]. Retinal detachment is a rarer complication of PE. We found that the difference in the number of pulmonary oedema and retinal detachment between groups was significant, similar to the findings by Kim et al. [10]. This is probably due to the much lower mean serum albumin in the massive proteinuria group, resulting in low colloid osmotic pressure, which causes fluid accumulation.

The mean birth weights in the nephrotic and non-nephrotic range groups were 1.42 kg and 2.11 kg, respectively, in our study, in concordance with studies by Tanacan et al. and Mateus J et al. i.e. 1.4 kg in the massive proteinuria group and 1.5 kg in the 5-9.9 g/24-h proteinuria group [2,11]. Individual adverse perinatal outcomes like stillbirth, NICU admission, SGA, need for respiratory support, neonatal seizures, and neonatal mortality were significantly higher in the nephrotic range group compared to the non-nephrotic range group in our study. Similar to our study, the difference in the rate of SGA between the two groups was statistically significant in the study by Bramham et al. [17]. NICU admissions were significantly higher in the nephrotic range proteinuric group, as noted in other previous studies [14,17]. A significantly higher number of stillbirths and FGR was seen in the higher proteinuria range group [9,18]. Nephrotic range proteinuria and its relation with early-onset PE with severe features increase the risks of adverse perinatal outcomes due to iatrogenic prematurity.

Our study's strength is its prospective recruitment and close monitoring of women for all the core outcomes set for PE [13]. We included only women with preeclampsia diagnosed according to ACOG 2020 criteria to capture all the new-onset hypertension and proteinuria. Also, we utilised the standardised quantitative definition of nephrotic range proteinuria [7]. The neonates were followed up for one month postnatally, and data on neonatal deaths are also available.

The limitations of our study were the relatively small sample size in the nephrotic range group and the single-centre experience. Also, our study did not have follow-up data of the women to assess the resolution or persistence of proteinuria in their postpartum period to rule out de novo renal diseases in pregnancy.

Further, we recommend future research with population-based multicentric studies with larger sample sizes. Also, with a focus on early onset PE and proteinuria, with postpartum follow-up.

## Conclusions

The study results highlight that nephrotic range proteinuria is associated with significantly higher adverse pregnancy outcomes, irrespective of the gestational age at diagnosis. It necessitates the utilisation of nephrotic range proteinuria to categorise the severity of the disease and its utilisation as a predictive tool for risk stratification among women with pre-eclampsia. It will also aid in strategic management by early termination of pregnancy to optimise maternal and fetal outcomes.
